# Comparison of two-view versus single-view digital breast tomosynthesis and 2D-mammography in breast cancer surveillance imaging

**DOI:** 10.1371/journal.pone.0256514

**Published:** 2021-09-29

**Authors:** Andria Hadjipanteli, Petros Polyviou, Ilias Kyriakopoulos, Marios Genagritis, Natasa Kotziamani, Demetris Moniatis, Anne Papoutsou, Anastasia Constantinidou

**Affiliations:** 1 Medical School, Shacolas Educational Centre for Clinical Medicine, Palaios dromos Lefkosias Lemesou, University of Cyprus, Aglantzia, Nicosia, Cyprus; 2 Bank of Cyprus Oncology Centre, Strovolos, Nicosia, Cyprus; 3 German Oncology Center, Agios Athanasios, Limassol, Cyprus; 4 Protypo Diagnostiko Kentro A. Kanakas & I. Kyriakopoulos, Nicosia, Cyprus; 5 The Breast Center of Cyprus, Karyatides Business Centre, Strovolos, Nicosia, Cyprus; 6 Breast Imaging Center, Limassol, Cyprus; 7 Ygia Polyclinic Private Hospital, Limassol, Cyprus; 8 Cyprus Cancer Research Institute (C.C.R.I.), Aglantzia, Nicosia, Cyprus; Northwestern University Feinberg School of Medicine, UNITED STATES

## Abstract

**Purpose:**

Limited work has been performed for the implementation of digital breast tomosynthesis (DBT) in breast cancer surveillance imaging. The aim of this study was to investigate the differences between two different DBT implementations in breast cancer surveillance imaging, for patients with a personal history of breast cancer.

**Method:**

The DBT implementations investigated were: (1) 2-view 2D digital mammography and 2-view DBT (2vDM&2vDBT) (2) 1-view (cranial-caudal) DM and 1-view (mediolateral-oblique) DBT (1vDM&1vDBT). Clinical performance of these two implementations was assessed retrospectively using observer studies with 118 sets of real patient images, from a single imaging centre, and six observers. Sensitivity, specificity and area under the curve (AUC) using the Jack-knife alternative free-response receiver operating characteristics (JAFROC) analysis were evaluated.

**Results:**

Results suggest that the two DBT implementations are not significantly different in terms of sensitivity, specificity and AUC. When looking at the two main different lesion types, non-calcifications and calcifications, and two different density levels, no difference in the performance of the two DBT implementations was found.

**Conclusions:**

Since 1vDM&1vDBT exposes the patient to half the dose of 2vDM&2vDBT, it might be worth considering 1vDM&1vDBT in breast cancer surveillance imaging. However, larger studies are required to conclude on this matter.

## Introduction

Digital breast tomosynthesis (DBT) has been under consideration for its use in breast screening alone or in combination with 2D digital mammography (DM) and/or synthetic mammography (SM) for several years. A significant number of clinical observer studies, including large clinical trials, have investigated the optimum implementation of DBT in breast screening [1–8].

There is a strong agreement that the introduction of DBT in breast cancer screening together with DM can provide several advantages, including increased specificity [5,9], reduced recall rate [6,10–17] and an increased cancer detection rate (although these increases are not statistically significant in all studies) [5,6,13,16,18–20]. Studies on DBT have shown that 2-view DBT (craniocaudal, CC and mediolateral oblique, MLO) might have a lower threshold detectable mass diameter [21,22] compared to 2-view DM, even though there is still an uncertainty on the diagnostic capabilities of DBT in calcifications detection [23–26]. The introduction of the two views (CC and MLO) of DBT together with the two views of DM (CC and MLO) in breast cancer imaging has some disadvantages too, including the increased interpretation time and image storage capacity, and at least double the radiation dose of that of DM [27]. The dose should ideally be kept as low as reasonably practical [28].

One solution to the increased patient dose, image interpretation time and storage capacity could potentially be the replacement of DM by SM [29,30] as it has been shown that SM is at least non inferior to DM. As more extensivity discussed in Hadjipanteli et al [31] several studies, carried out after Hologic SM was approved by FDA, support that the performance of SM is least equal in terms of sensitivity (43,44), specificity (44,45), AUC (97), cancer detection rate (97) and recall rate (100–103) However, work is still undertaken on the optimal utilization of SM [32] and there is therefore uncertainty on its use in screening. Furthermore, not all hospitals and breast imaging centres have the immediate financial capacity to get access to SM, as it requires at least a specialised image analysis software, which forms an additional separate cost to tomosynthesis and cannot always be easily justified.

Alternatively, to decrease patient dose, image interpretation time and storage capacity, work has been undertaken for the assessment of one- or two-views DM together with one- or two-views DBT in breast cancer screening [1,5]. Under the hypothesis that 1-view CC DM with 1-view MLO DBT has an equal diagnostic accuracy to 2-view DM with 2-view DBT, then this could serve as an alternative solution to the disadvantages of DM together with DBT in screening. This solution has been considered in screening population but this current study investigates its utility in breast cancer surveillance imaging.

Limited work has been performed for the implementation of DBT in breast cancer surveillance imaging [13,33] which involves the imaging of patients that have a past personal history of breast cancer. Margolies *et al* (2013) have shown that for patients with an elevated risk of breast cancer due to family history, personal history of breast cancer or prior biopsy (e.g. for atypia, LCIS, or multiple biopsies), there is no detectable incremental benefit for DBT, compared to DM [13]. Sia *et al* (2016) evaluated the rate of indeterminate findings (number of lesions requiring additional imaging studies for clarification) of DBT with DM, and DM alone, using images of patients that underwent routine surveillance following breast cancer treatment [33]. They showed that the addition of DBT to DM reduces indeterminate findings. Further studies are required for better understanding the demands of breast cancer surveillance and the input DBT can have in it.

Ramani *et al* (2011) in a pictorial review discusses how the normal breast may be altered post-operation, and supports that DBT adds incremental value in surveillance imaging [34]. Changes in the breast after breast conserving therapy, which include breast oedema and fibrosis, seroma collection, architectural distortion and calcifications, and various reconstruction techniques, may impact on the performance of breast imaging by limiting compressibility of the breast and by mimicking or hiding tumour recurrence [35,36]. In addition, a mastectomy and thus the non-existence of a contralateral image, for back-to-back comparison with the ipsilateral image, might also impact on the performance of breast imaging of surveillance population, in comparison to imaging screening population, where there is availability of both breast images. The above factors might make patients with breast cancer history a distinct group [18,37], and thus breast cancer surveillance imaging implementation might require independent optimization. More specifically, with the hypothesis that there might be a reduced sensitivity and specificity of when treated breast is imaged with DM alone, assessment of the benefit of different DBT implementation methods in surveillance imaging is required.

The aim of this study was to assess the implementation of a single-view DBT in breast cancer surveillance imaging for patients that have a personal history of breast cancer as it has not been studied before, and could reduce the patient radiation dose, image interpretation time and storage capacity. The single-view implementation method comprised of one view (CC) in DM and the opposite view (MLO-view) in DBT. This is done to achieve wider breast image coverage. The MLO view was selected for DBT as it enables inclusion of more breast tissue than the CC position [4], and in this way a better use of the 3D nature of the DBT can be achieved. The single-view DBT implementation (which will be referred to as 1vDBT&1vDM) was compared to the standard 2-view DBT ‘combo-mode’ (CC DM, MLO DM, CC DBT, MLO DBT; which will be referred to as 2vDM&2vDBT), using observer studies. Conclusions were drawn on the comparison of the two implementations that could provide guidance to imaging cancer centres. As discussed above, the single-view implementation could be of particular interest to breast centres that do not have access, due to financial reasons or else, to synthetic imaging.

## Methods

### Patient cohort

This study was approved by the Cyprus National Bioethics Committee. The patient cohort was from a single centre, the Bank of Cyprus Oncology Center (BOCOC, Nicosia, Cyprus). Surveillance imaging at the Centre is offered to any breast cancer patient who undergoes breast cancer treatment including breast conserving surgery or mastectomy with or without post-operative radiotherapy and systemic therapy.

Surveillance imaging at the BOCOC is carried out annually using the commercial DBT system, Hologic Selenia Dimensions (Hologic Inc., Bedford, MA, USA), using the 2-view (or ‘combo’) mode: for each patient 2-view (CC and MLO) DM and 2-view (CC and MLO) DBT images are acquired. Fifteen projections over an angular range of 15° are acquired and reconstructed using filtered back projection.

The patient inclusion criteria of this study included (i) women aged 18 years old or above, (ii) who attended mammography surveillance imaging (before the commencement, and independently of this study) at the BOCOC between September 2014 and December 2017 under the local mammography surveillance imaging protocol described above, (iii), following breast cancer treatment at the same centre. Patients with breast implants were excluded from the patient cohort.

### Image sets

For the requirement of this study three different groups of patients were required: (A) normal, (B) biopsied benign and (C) biopsied malignant. Normal cases (group A) included a random selection of patients from the mammography surveillance population that satisfied the inclusion criteria described above and did not have any symptoms or signs of breast cancer until at least one year after the acquisition of their images that were used in this study. The biopsied benign group (group B) included all the patients that met the inclusion criteria for the study and following surveillance imaging at the BOCOC they were interpreted as normal through histopathology. All had at least one year of malignancy-negative imaging follow-up [1,5,7]. The biopsied malignant group (group C) included all the patients that met the inclusion criteria for the study and following surveillance imaging at BOCOC they were found to have mammographic abnormalities interpreted as malignant through histopathology.

For each patient two groups (for the two DBT implementation methods tested) of fully anonymised images were prepared before data was further processed. The requirement of informed consent was not waived by the National Bioethics Committee, however, patient consent was still acquired by the BOCOC from its patients. The two groups of images from all patients were then separated in two sets that would be assessed by the observers. In order to minimize learning bias acting in favor of one implementation method, the first set included half of the patient cases from one DBT implementation and half of the cases from the other DBT implementation. The second set included the opposite halves. A patient case was never presented twice in one set.

The reference standard (ground truth) of the patient cases used, including the absence of lesions in the breast (for group A) or lesion location, lesion type, and histopathology confirmation (for groups B and C), was defined using the histopathology and radiological case reports from clinical routine. The radiological case reports were prepared by three radiologists who had knowledge of each patient’s background and access to additional imaging tests including priors and histopathology reports of each patient that participated in the study.

### Observers’ studies

Six observers-radiologists participated in the study, from five different breast cancer centres within Cyprus, none of who had access to the radiological case reports. The radiologists had a median of 11–25 years of experience in DM and a median of 4–6 years of experience in DBT. None of the radiologists had experience in reading images using the 1vDBT&1vDM method.

All observer studies were performed under clinical conditions, using dual 5-MP mammographic displays and RadiANT PACS-DICOM (radiantviewer.com) and Weasis Medical Viewer (https://nroduit.github.io/en/) as viewers.

The two sets of images were shown to each observer in two sessions, with two weeks gap in between. Before the initiation of the study, all observers underwent training on the observers study that they would need to carry out, using the same viewer, tools and a set of images from 14 different patients that were acquired using the Hologic system; half were malignant and half were benign cases; half were presented in the 1vDBT&1vDM mode and half were presented in the 2vDBT&2vDM mode. The observers were blinded to any information about the patient and any prior imaging, but they were aware of the purpose of the study.

The observers assessed each patient separately and annotated their results on specifically designed patient forms. For each patient case each observer assigned a BI-RADS® score (1—normal, 2—benign finding, 3 –low probability of malignancy, 4 –suspicious of malignancy, 5 –highly suspicious of malignancy) as established by the American College of Radiology. For each finding each observer gave a level of suspiciousness score on a 6-point arbitrary scale. Using this the observers had to answer the question on how suspicious they are that the finding is suspicious of malignancy (1 –no, very confident, 2 –no, moderately confident, 3 –no, slightly confident, 4 –yes, slightly confident, 5 –yes, moderately confident, 6 –yes very confident). For each finding lesion location was also recorded on the patient’s form, which included two breast diagrams, one in CC and one in MLO direction. The observers had to choose one or more of the nine subdivided regions that were marked on the breast diagram, as an approximation to where they have identified one or more lesions. For DBT, the slice number in which the finding was observed was also registered.

### Statistical analysis

The BI-RADS® categorisation of the patient cases by the six observers was used to calculate sensitivity (true positive rate) and specificity (true negative rate) for each of the observers and for each of the DBT implementations, thus having between-subjects independent variables. BI-RADS® category 3 or higher was defined as a positive interpretation, while category 1 and 2 were defined as negative interpretation. Cases with biopsied benign lesions were considered as false positives, if the readers rated them positive.

Average sensitivity and specificity for the two DBT implementation methods were calculated using a generalized linear model (GLM) to account for multiple reader and multiple case repeated measures. Modality and readers were used as factors in preparing the GLM model. Model-based 95% confidence intervals were also calculated. The statistical significance between the two different DBT implementation methods for each reader separately was estimated using McNemar’s paired test, as recommended by Lu *et al* [38]. The threshold for significance used was p-value<0.05.

The lesions localisations and the level of suspiciousness as recorded by the observers were used in the Jack-knife alternative free-response receiver operating characteristics (JAFROC) analysis [39]. It is worth noting that the BI-RADS® scores were not used in this analysis as a receiver operating characteristics analysis requires the diagnostic confidence to be expressed in an ordinal scale [40] and BI-RADS® scale is not considered ordinal. JAFROC analysis provides a figure of merit (FoM) defined as the probability that a correctly marked lesion is rated higher than the highest-rated mark on a normal or benign case [41]. The FoM represents the area under the curve (AUC) of the lesion localisation fraction *versus* the non-lesion localisation fraction. The registration of the finding was considered correct if the lesion mark made by an observer was within the actual lesion region (one of nine subdivided breast regions as defined by the radiological case report and biopsy from clinical routine), and for DBT within ±3 slices from the actual DBT slice. As elsewhere [1], JAFROC analysis was also carried out separately for each lesion type; non-calcification or calcification (if a lesion was composed of both types, it was counted in each category), as well as for two density categories (low: a and b, high: c and d, according to BI-RADS® 5^th^ Edition, as obtained from the radiological case reports from clinical routine).

Statistical significance analysis was carried out for JAFROC analysis results using the Dorfman-Berbaum-Metz analysis of variance. Random-reader and random-case analysis were performed for JAFROC analysis. The above analyses were performed using the open-access JAFROC software by Dev Chakraborty (version 4.2.1, DevChakraborty.com).

### Mean glandular dose

Finally, the mean glandular doses were retrieved from the DICOM (Digital Imaging and Communications in Medicine) headers of the images. The mean glandular doses were used for the comparison of the average dose per single breast (total dose of all views undergone observation for a single breast) between the two different DBT implementations tested.

## Results

### Patient cohort and characteristics

Within the patient cohort that met the study’s inclusion criteria 24 biopsied benign (group B) patients and 57 biopsied malignant (group C) patients were identified. For group A (normal), 37 women were randomly selected from the patient cohort that met the study’s inclusion criteria. These numbers were estimated to be appropriate based on the study by Obuchowksi *et al* 2000 on required sample size for receiver operating characteristic studies, according to the following assumptions [42]: 6 observers, small to moderate variability among the observers, moderate level of accuracy of the test, small difference in accuracy between imaging techniques, and a ratio of patients without, to patient with history of breast cancer, of approximately 1:1, which were all expected to be satisfied in this study. In total, 118 sets of DM and DBT breast images from 118 female patients (median age 65, range 41–88 years old) imaged at BOCOC were reviewed by the observers.

One set of images could include two or four images for the 1vDM&1vDBT implementation and four or eight images for the 2vDM&2vDBT implementation, depending on whether the participant had undergone mastectomy. Out of the 118 cases included in the study, 54 had undergone a mastectomy, therefore for them only the ipsilateral image was available. For 64 cases, both ipsilateral and contralateral images were available. In total, 64 breasts out of the (sum of 64, 64 and 54) 182 breasts assessed by the observers had undergone treatment and (sum of 64 and 54) 118 had not. The total number of images reviewed by each observer was 1092. Tables 1 and 2 show details of the patient cases that were included in this study.

**Table 1:** Details of the patient cases included in the observers study.

**Patient group**	**Number of cases**
Normal (group A)	37 (31%)
Biopsied benign (group B)	24 (20%)
Biopsied malignant (group C)	57 (48%)
**Patient age**	**Age (years)**
Median age (range)	65 (41–88)
40–49	5 (4%)
50–59	24 (20%)
60–69	49 (42%)
70–79	31 (26%)
≥80	9 (8%)
**BI-RADS breast density**	**Number of cases**
Almost entirely fatty (a)	36 (31%)
Scattered fibroglandular densities (b)	32 (27%)
Heterogeneously dense (c)	30 (25%)
Extremely dense (d)	20 (17%)

**Table 2:** Details of lesion cases of group C (malignant) included in the observers study.

**Lesions in patients**	**Number of biopsied lesions**
Patients with one malignant lesion	49
Patients with two malignant lesions	7
Patients with three malignant lesions	3
**Lesion type**	**Number of observed lesions**
Non-calcified malignant	48
With calcifications malignant	26
Patients with one malignant lesion	49

Within group C, 49 patients had one malignant lesion; 7 patients had two malignant lesions (total of 14 lesions) and one patient had 3 malignant lesions, giving a total of 66 malignant lesions. 31 patients were classified as low breast density and 26 patients were classified as high density.

### Sensitivity and specificity

**Fig 1:**
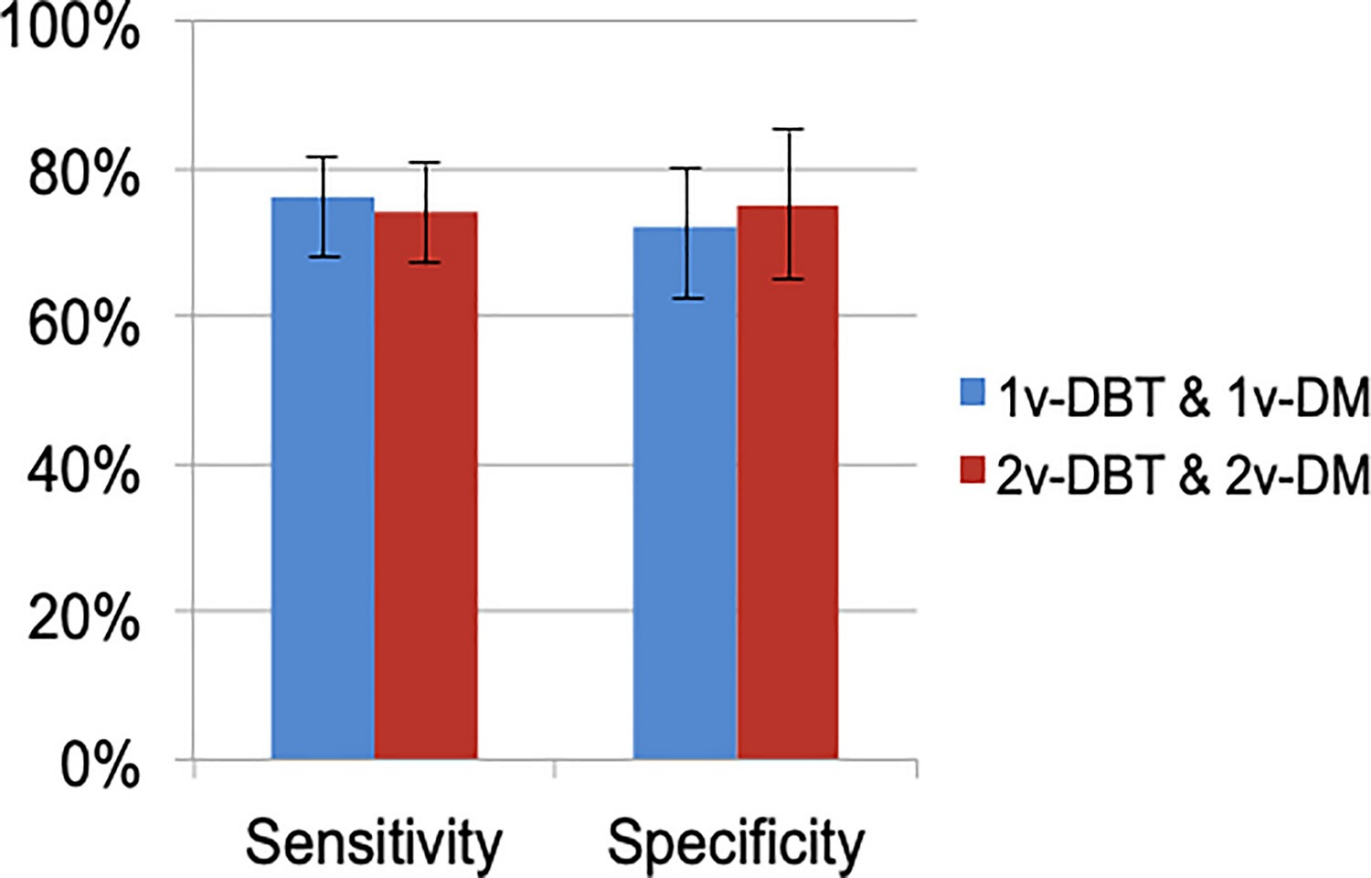
Average sensitivity (%) and specificity (%) on each DBT implementation method. The error bars indicate 95% confidence intervals.

The sensitivity and specificity per observer and on average are summarised in Table 3. The sensitivity range (and average sensitivity) of 1vDM&1vDBT and 2vDM&2vDBT was 67%-81% (average 76%) and 67%-84% (average 74%) respectively (Fig 1). The specificity range (and average specificity) of 1vDM&1vDBT and 2vDM&2vDBT was 68%-84% (average 72%) and 59%-90% (average 75%) respectively. The use of the additional views in DM and DBT yielded a slight (non-significant) increase in sensitivity for four of the six observers, and a decrease for the other two observers. For one of these two observers the difference in sensitivity was statistically significant. Four of the six observers performed better in terms of specificity with the addition of the second view of DM and DBT, but two of them presented a decreased specificity. For two of the observers the specificity changed significantly with the addition of the second view of DM and DBT. On average, no statistically significant differences were found between the two DBT implementations either for sensitivity (p = 0.63) or specificity (p = 0.67).

**Table 3:** Sensitivity and specificity for the average of all readers, using the BI-RADS® score of the most suspicious finding on each case.

Reader	1vDM&1vDBT	2vDM&2vDBT
Sensitivity(%)		
R1	67	67
R2	81	82
R3	79	68*
R4	77	70
R5	81	84
R6	68	70
All	76	74
Specificity(%)		
R1	82	87
R2	68	70
R3	80	92*
R4	84	74*
R5	74	69
R6	61	59
All	72	75

*Significant (p-value < 0.05) difference of 2vDM&2vDBT with respect to 1vDM&1vDBT.

### JAFROC analysis

Table 4 shows the individual JAFROC FoM per reader. JAFROC FoM varied in the range 0.634–0.805 (average 0.757) and 0.677–0.793 (average 0.751) between observers for 1vDM&1vDBT and 2vDM&2vDBT respectively.

**Table 4:** JAFROC figure of merit (FoM) per reader and on average.

Reader	1vDMT&1vDBT	2vDM&2vDBT
R1	0.770	0.790
R2	0.782	0.793
R3	0.805	0.754
R4	0.775	0.743
R5	0.779	0.751
R6	0.634	0.677
All	0.757	0.751

**Fig 2:**
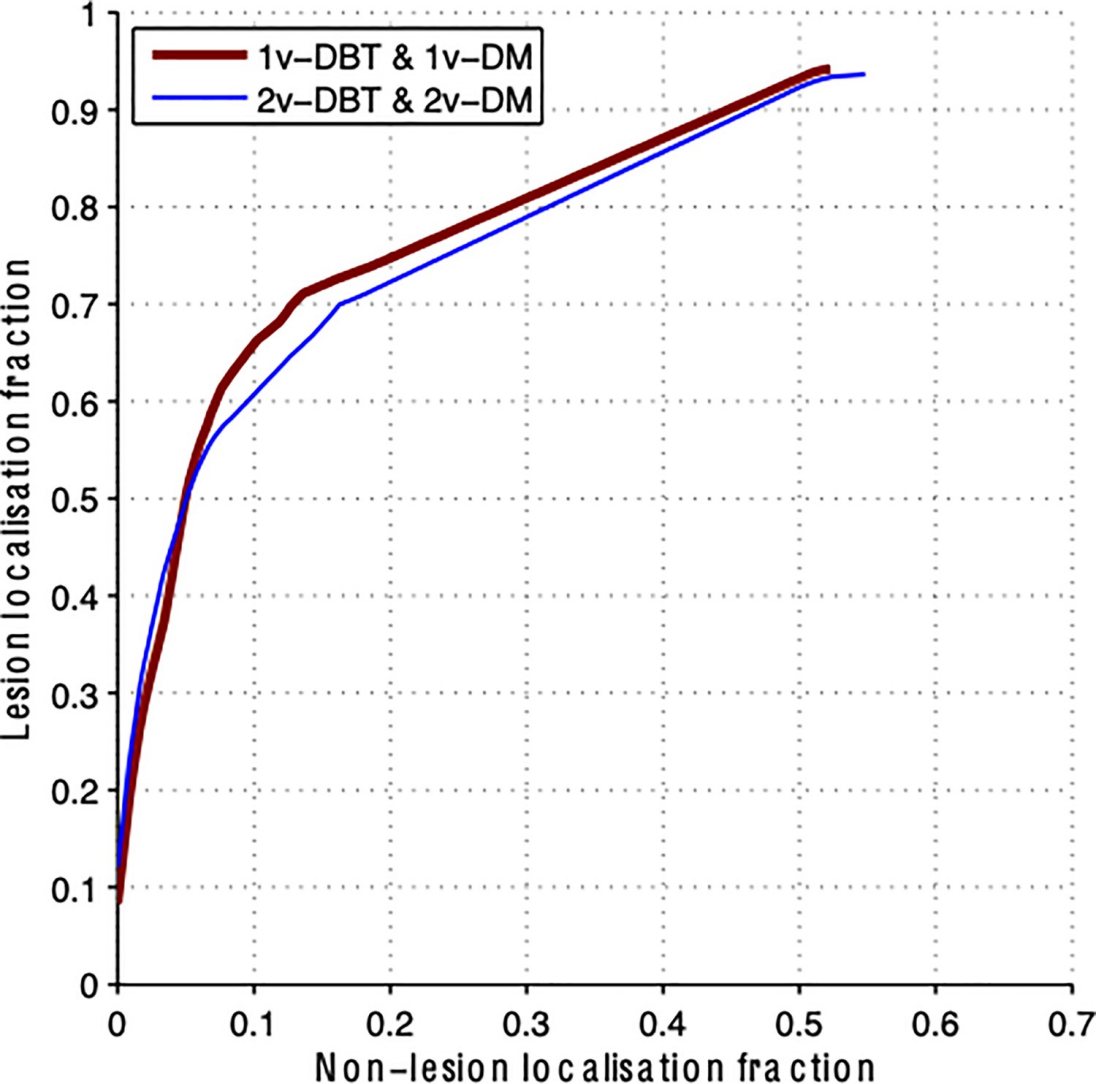
JAFROC analysis averaged for all readers for the two DBT implementations assessed: 1vDMT&1vDBT in red and 2vDMT&2vDBT in blue. The lesion localization fraction is the number of correctly identified lesions divided by the total number of lesions and the non-lesion localization fraction is the number of marks which are not close to any lesions, divided by the total number of images.

Fig 2 shows the JAFROC curve averaged for all readers. The FoM for 1vDBT&1vDM was similar to 2vDBT&2vDM and no statistically significant difference was found, either on average (p = 0.716) or per reader (p = 0.19–0.64).

Using JAFROC analysis, no significant difference was found between the two implementation methods either when separating the patient cases by breast density (low, p = 0.271; or high, p = 0.137) or when separating the lesions by type (non-calcifications, p = 0.562; or calcifications, p = 0.325), showing that 1vDBT&1vDM is similar to 2vDBT&2vDM independent of breast density or lesion type

### Mean glandular dose

Following the reporting the mean glandular dose per breast, the average mean glandular dose per DBT implementation among 182 breasts were the following: (i) 2vDM&2vDBT: 8.10±3.93 mGy and (ii) 1vDM&1vDBT: 3.95±1.38 mGy. As expected 1vDM&1vDBT exposes each breast to approximately half the dose of that of 2vDM&2vDBT.

## Discussion

This retrospective clinical observer study assessed a possible single-view DBT implementation (1vDM&1vDBT) for breast cancer surveillance imaging and compared to the standard 2vDM&2vDBT. Comparison of the implementation methods was made in terms of sensitivity, specificity and JAFROC FoM. Results suggest that the two DBT implementations are not significantly different. When looking separately at the two main different lesion types, calcifications and non-calcifications, and two different density levels, we found that there is not a statistically significant difference in the performance of the two DBT implementations either.

Based on the results of this study, it can be concluded that the use of 1vDBT&1vDM for breast cancer surveillance imaging may be feasible, since the added value of the second DBT and DM views in terms of the metrics assessed was not found to be significant. The use of 1vDM&1vDBT can be advantageous over 2DM&2vDBT in clinical practice, especially for breast centres that do not have access to SM yet due to financial reasons (as it requires at least a specialised image analysis software, which forms an additional separate cost to tomosynthesis and cannot always be easily justified) or other reasons. 1vDM&1vDBT can be advantageous over 2DM&2vDBT due to the reduced radiation dose, image acquisition and interpretation time, and image storage capacity. As shown, the combination of two views of DBT to two views of DM would at least double the radiation dose when compared to 1vDM&1vDBT. The additional dose should be justified by a better performance and an increased diagnostic benefit, but still keeping the dose as low as reasonably possible is required [28].

As more extensively presented in Hadjipanteli et al 2019 [31], the combination of 1vDBT or 2vDBT with 1vDM or 2vDM was investigated in several studies [1,2,4,43–46], with the difference that its implementation was in breast cancer screening and not surveillance imaging. Rafferty *et al* [4] showed that using Hologic 2vDM, 2vDM&1vDBT, and 2vDM&2vDBT the AUCs were 0.828, 0.864, and 0.895 respectively. Compared with DM, the AUC increased by 0.036 (p = 0.009) with the addition of 1vDBT and increased by 0.068 (p < 0.001) with the addition of 2vDBT. The addition of 2vDBT resulted in higher AUC than the addition of 1vDBT (Δ AUC 0.032, p = 0.021); however, this difference did not meet their criterion of significance (p<0.0167), similar to the current study. It is worth noting that a significant reduction in recall rate, which might be an important metric, was found in the aforementioned study when using 1vDBT&2vDM *versus* 2vDBT&2vDM. It is also worth noting that the manufacturer does recommend using both views of DM and DBT (Hologic, Spring, 2015). With regards to the use of 1vDBT of Siemens (Siemens, Erlangen, Germany), provided the user has appropriate training, it has been found that DBT MLO view-only is not inferior to 2vDM or 2vDM&2VDBT and might be feasible for breast cancer screening, which agrees with the conclusions of this study [1,2]. Studies using General Electric (GE) single-view combinations of DBT alone or with DM, have shown that its sensitivity is non-inferior to DM, also in agreement with the conclusions of this current study. To the best of our knowledge no study compared directly 1vDM&1vDBT versus 2vDM&2vDBT for surveillance imaging, therefore a direct comparison of our results to other studies is not currently possible.

This study is one of the very few studies that assess DBT implementation methods on breast cancer surveillance imaging. All data was collected from a single cancer centre and the use of a true DM and DBT screening population and clinical DBT imaging protocol was made. DBT was already clinically implemented in surveillance imaging at the centre of data collection of this study, which forms an advantage over studies where the sample used was from a participants’ cohort that attended DBT imaging for further assessment of possible abnormalities, following DM screening. Therefore there was no bias on sample selection. Sample cases were selected based on availability only and no patient selection criteria, for example inclusion of only small-sized nodules or highly dense breasts.

One limitation of our study forms the fact that the patient history and patient previous images were not provided to the observers, and this might have an impact on the performance of the study. This was mainly due the unavailability of previous images for 1/3 of the patients cohort (September 2014- September 2015 patients cohort), as before then surveillance imaging was only carried out using DM. Our study therefore, does not fully represent a clinical case scenario. One might expect though that if patient history and previous images were provided to the observers, would cause a similar effect to the two implementations assessed. Therefore the relative comparison of the two implementations might have not been affected significantly with the absence of patient history and previous images from the study. There was a time interval of two weeks between sessions, similar to other studies [8,47–50]. To limit further a potential bias due to readers’ short-term memory, the cases were displayed in a random order [51]. Finally, even though the number of cases used fit within the statistical analysis criteria set for this type of studies [42], the sample size is relatively small–due to availability–and a larger study would add more confidence to the conclusions of this study.

In conclusion, the need to keep radiation dose as low as reasonably applicable requires the usage of methods that can provide high diagnostic quality at a justified dose. This study shows that the diagnostic quality in terms of sensitivity, specificity and AUC of JAFROC analysis of 1vDM&1vDBT and 2vDM&2vDBT appear to be equivalent. Since 1vDM&1vDBT exposes the patient to half the dose of 2vDM&2vDBT, it might be worth considering 1vDM&1vDBT in breast cancer surveillance. Larger studies are required to conclude on this matter.

## Abbreviations

DBT: Digital breast tomosynthesis
DM: 2D-mammography
SM: synthetic mammography
CC: craniocaudal
MLO: mediolateral oblique
BOCOC: Bank of Cyprus Oncology Centre
JAFROC: Jack-knife alternative free-response receiver operating characteristics
AUC: area under the curve
FOM: figure of merit
1vDM&1vDBT: 1-view digital breast tomosynthesis with 1-view digital mammography
2vDM&2vDBT: 2-view digital breast tomosynthesis with 2-view digital breast tomosynthesis
